# Fueling Inflamm-Aging through Mitochondrial Dysfunction: Mechanisms and Molecular Targets

**DOI:** 10.3390/ijms18050933

**Published:** 2017-04-28

**Authors:** Anna Picca, Angela Maria Serena Lezza, Christiaan Leeuwenburgh, Vito Pesce, Riccardo Calvani, Francesco Landi, Roberto Bernabei, Emanuele Marzetti

**Affiliations:** 1 Department of Geriatrics, Neuroscience and Orthopedics, Catholic University of the Sacred Heart School of Medicine, 00168 Rome, Italy; anna.picca1@gmail.com (A.P.); francesco.landi@unicatt.it (F.L.); roberto.bernabei@unicatt.it (R.B.); emarzetti@live.com (E.M.); 2 Department of Biosciences, Biotechnology and Biopharmaceutics, University of Bari, 70125 Bari, Italy; angelamariaserena.lezza@uniba.it (A.M.S.L.); vito.pesce@uniba.it (V.P.); 3 Department of Aging and Geriatric Research, Institute on Aging, Division of Biology of Aging, University of Florida, Gainesville, FL 32611, USA; cleeuwen@ufl.edu

**Keywords:** mitophagy, sterile inflammation, mitochondrial biogenesis, mitochondrial dynamics, TFAM, mitochondrial quality control (MQC), inflammasome, damage-associated molecular patterns (DAMPs)

## Abstract

Among the complex determinants of aging, mitochondrial dysfunction has been in the spotlight for a long time. As the hub for many cellular functions, the maintenance of an adequate pool of functional mitochondria is crucial for tissue homeostasis. Their unique role in energy supply makes these organelles essential, especially in those tissues strictly dependent on oxidative metabolism. Mitochondrial quality control (MQC) is ensured by pathways related to protein folding and degradation as well as by processes involving the entire organelle, such as biogenesis, dynamics, and mitophagy. Dysfunctional MQC, oxidative stress and inflammation are hallmarks of senescence and chronic degenerative diseases. One of the consequences of age-related failing MQC and oxidative stress is the release of mitochondria-derived damage-associated molecular patterns (DAMPs). Through their bacterial ancestry, these molecules contribute to mounting an inflammatory response by interacting with receptors similar to those involved in pathogen-associated responses. Mitochondrial DAMPs, especially cell-free mitochondrial DNA, have recently become the subject of intensive research because of their possible involvement in conditions associated with inflammation, such as aging and degenerative diseases. Here, we review the contribution of mitochondrial DAMPs to inflammation and discuss some of the mechanisms at the basis of their generation.

## 1. Introduction

Aging is a complex and multi-factorial process characterized by increased risk of adverse health outcomes [1]. Understanding the intimate mechanisms of aging is therefore instrumental for contrasting its negative correlates [1]. As initially proposed in the “mitochondrial theory of aging”, mitochondria are deeply involved in the aging process mainly through respiratory dysfunction and oxidant generation [2,3]. Although unique as fueling systems within the cell, mitochondria participate in other essential functions, including heme metabolism, regulation of intracellular calcium homeostasis, modulation of cell proliferation, and integration of apoptotic signaling [4,5,6,7]. It is therefore crucial that a pool of healthy and well-functioning organelles is maintained within the cell. To this aim, a comprehensive set of adaptive quality control processes operates via interrelated systems, including pathways pertaining to protein folding and degradation, mitochondrial biogenesis, dynamics, and autophagy (mitophagy) [8,9]. The activation of individual MQC pathways depends on the degree of mitochondrial damage. Due to these vital responsibilities, disruption of the MQC axis is invoked as a major pathogenic mechanism in a number of disease conditions (i.e., cancer, cardiovascular disease, diabetes, and neurodegenerative disorders) and aging [8,10,11].

Together with mitochondrial dysfunction, chronic inflammation is another hallmark of both aging and degenerative diseases [12]. Interestingly, emerging evidence suggests that the two phenomena are related to one another. In particular, circulating cell-free mitochondrial DNA (mtDNA), one of the cell damage-associated molecular patterns (DAMPs), has been proposed as a functional link between mitochondrial damage and systemic inflammation [13,14]. Indeed, mtDNA, which is released as a result of cellular stress, contains hypomethylated CpG motifs resembling those of bacterial DNA and is therefore able to induce an inflammatory response [15]. These regions bind and activate membrane or cytoplasmic pattern recognition receptors (PRRs), such as the Toll-like receptor (TLR), the nucleotide-binding oligomerization domain (NOD)-like receptor (NLR) [15], and the cytosolic cyclic GMP-AMP synthase (cGAS)-stimulator of interferon genes (STING) DNA sensing system-mediated pathways [16]. The mechanisms responsible for the generation of mitochondrial DAMPs as well as their contribution to the inflammatory *milieu* that characterizes aging and its associated conditions are not completely understood.

Here, we provide an overview of major processes of MQC and their changes during aging. Subsequently, we describe candidate mechanisms responsible for generating and releasing mitochondrial DAMPs. Finally, we summarize the current evidence in support of mitochondrial DAMPs as triggers for age-related chronic inflammation.

## 2. MQC Processes

### 2.1. Mitochondrial Proteolytic Quality Control System

The mitochondrial proteolytic quality control system consists of subcompartment-specific proteases (mitoproteases) and the ubiquitin-proteasome system (UPS) that together regulate mitochondrial protein turnover and degrade misfolded or oxidized proteins [17]. Mitochondrial proteases act as the first line of defense against mild mitochondrial damage [8]. Within the mitochondrial matrix, protein turnover is controlled by 3 AAA proteases: the soluble Lon and ClpP and the membrane-bound m-AAA [18]. In the inter-membrane space, mitochondrial protein quality is ensured by the membrane-bound i-AAA Yme1L1, the soluble HtrA2/Omi, the metallopeptidases OMA1, and the presenilins-associated rhomboid-like protein (PARL) [8]. These mitoproteases can also affect mitochondrial fate and cell viability by acting on mitochondrial dynamics, mitophagy and apoptosis [19,20].

Mitochondrial protein turnover is also regulated by the cytosolic UPS [21]. A proteomic study in murine heart identified numerous proteins to be ubiquitinated in the various mitochondrial compartments [22]. However, the mechanism whereby the cytosolic proteasome degrades integral mitochondrial membrane proteins is still a matter of debate.

Similar to the endoplasmic reticulum (ER), mitochondria possess a stress responsive system for protein degradation that shares with its ER analogue some key components, including the AAA ATPase p97 and the cofactor Npl4 [23]. Under stress conditions, when protein degradation pathways become insufficient to restore a normal mitochondrial function, a retrograde signal is triggered, which acts on the nuclear genome. This pathway, named mitochondrial unfolded protein response (UPRmt) [24], up-regulates the expression of nuclear genes encoding mitochondrial stress proteins, including chaperonin 10 and 60, mtDnaJ, ClpP and Yme1 [24]. Through these mediators, the UPRmt promotes mitochondrial proteostasis by improving protein folding and degrading irreversibly damaged proteins.

### 2.2. Mitochondrial Biogenesis

**Figure 1:**
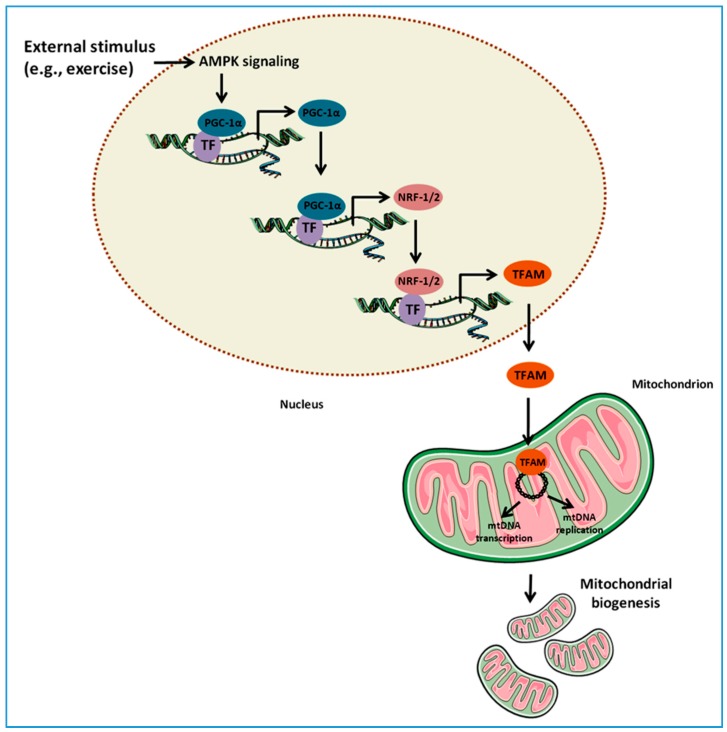
Schematic representation of the regulation of mitochondrial biogenesis. In response to external stimuli (e.g., skeletal muscle contraction or exercise), the nuclear genome coordinates the expression of nuclear and mitochondrial proteins. This pathway is triggered by the activation of signaling molecules, including AMP-activated protein kinase (AMPK), and converges on the expression of the transcriptional coactivator peroxisome proliferator-activated receptor gamma coactivator-1α (PGC-1α), the master regulator of mitochondrial biogenesis. PGC-1α promotes its own expression as well as that of the nuclear respiratory factor 1 and 2 (NRF-1/2). NRF-1 and 2 bind and up-regulate the expression of nuclear genes encoding mitochondrial proteins as well as the expression of mitochondrial transcription factor A (TFAM), which is subsequently transported into mitochondria. Here, TFAM binds to mitochondrial DNA (mtDNA) and activates the transcription and replication of the mitochondrial genome, a crucial step in the generation of new organelles. TF, transcription factor.

Mitochondrial biogenesis is a multi-stage process generating new organelles that, in a dynamic balance with their degradation, regulate the mitochondrial content within the cell. Mitochondriogenesis is orchestrated through the expression of nuclear and mtDNA-encoded genes. In particular, transcriptional coactivators belonging to the peroxisome proliferator activated receptor gamma coactivator-1 (PGC-1) family (e.g., PGC-1α and PGC-1β), the nuclear respiratory factor (NRF) 1 and 2, and the estrogen-related receptor α (ERRα), coordinate the expression of mitochondrial proteins encoded by nuclear DNA [25]. Subsequently, several mitochondrial proteins are expressed, including those binding the mtDNA (e.g., mitochondrial transcription factor A (TFAM), B1 and B2 (TFB1M and TFB2M)), which are then transported into mitochondria by a protein import machinery [26]. Once entered into the mitochondrion, mtDNA-binding proteins directly activate mtDNA transcription and replication (Figure 1).

Both qualitative and quantitative changes in mtDNA have been found with aging in various tissues and across species [27,28]. MtDNA is organized into protein-DNA complexes, called nucleoids, within the mitochondrial matrix [29]. One prominent component of such complexes is TFAM, which associates with the inner mitochondrial membrane [30]. TFAM is a member of the high-mobility-group (HMG) proteins, able to bind, unwind and bend mtDNA without sequence specificity, but with preferential interaction with some regions [31,32,33]. TFAM participates in several processes, including mtDNA replication and transcription, mtDNA maintenance, and possibly mtDNA repair [34,35,36]. Recently, studies employing in vivo binding analysis of TFAM to specific mtDNA regions have shown that the modulation of TFAM-mtDNA interaction regulates mitochondrial biogenesis [37]. Noteworthy, a dysregulation of this interaction, secondary to TFAM and/or mtDNA alterations, has been identified in aged tissues as a new mechanism modulating mitochondrial biogenesis during aging [37].

The importance of the modulation of TFAM binding also resides in its ability to stabilize the mtDNA. The latter, if unbound, becomes more fragile and prone to degradation, which ultimately leads to organelle dysfunction. The identification of misplaced TFAM and mtDNA in the plasma of persons with chronic inflammatory diseases suggests these molecules may be involved in the inflammatory response [14,38]. Whether defective TFAM binding to mtDNA is also responsible for mitochondrial DAMP generation and activation of the DAMP-sensing system during aging is presently unclear.

### 2.3. Mitochondrial Dynamics

**Figure 2:**
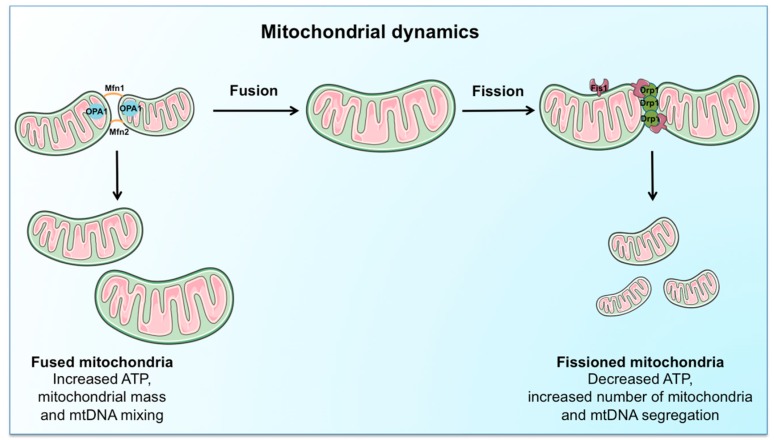
Schematic representation of the regulation of mitochondrial dynamics. The coordination of fusion and fission events is ensured by a complex machinery, involving mitofusin (Mfn) 1 and 2, optic atrophy protein 1 (OPA1), dynamin-related protein 1 (Drp1), and fission protein 1 (Fis1). Mitochondrial fusion, by interconnecting organelles, promotes mtDNA mixing and enhances bioenergetic efficiency. Mitochondrial fission, instead, ensures equal organelle segregation between daughter cells, reduces ATP generation, and targets defective mitochondria for their subsequent disposal via mitophagy.

Mitochondrial dynamics control organelle shaping, metabolic plasticity [39], redox signaling [40], and cell death/survival pathways [41] through the coordination of fusion and fission events. On the one hand, fusion allows for mitochondrial interconnection, favoring mtDNA mixing, signal transmission and exchange of metabolites within the network [42]. The process also serves to prevent focal accumulation of mutant mtDNA [42], thereby preserving mtDNA integrity, and to regulate oxidative metabolism [43]. On the other hand, mitochondrial fission ensures equal organelle segregation between daughter cells and targets defective mitochondria for their subsequent removal through mitophagy [44] (Figure 2).

The integration of mitochondrial dynamics and mitophagy ensures an efficient MQC process and contributes to preserving metabolic cellular “fitness” and plasticity [9]. Derangements of fusion-fission have been proposed as a mechanism contributing to the formation of structurally abnormal and dysfunctional mitochondria under stress conditions and during senescence [45].

The exposure of cultured cells to subcytotoxic doses of hydrogen peroxide represses the expression of fission protein 1 (Fis1), thereby promoting the formation of elongated mitochondria with increased oxidant emission [45]. Excessive activation of fission can also induce mitochondrial dysfunction. Indeed, mitochondrial fragmentation, down-regulation of fusion and impaired bioenergetics have been detected in muscle of diabetic persons [46]. On the other hand, depletion of the mitochondrial fusion factor optic atrophy protein 1 (OPA1) disintegrates the mitochondrial network and sensitizes cultured cells to apoptosis [47]. Conversely, blockade of Fis1 or dynamin-related protein 1 (Drp1) inhibits mitochondrial fragmentation and the execution of apoptosis in cell culture systems [47].

Imbalanced mitochondrial dynamics toward fission have been found in several disease conditions [48] as well as in age-related sarcopenia [49] and cancer cachexia [50]. Conversely, increased fusion in very old rats has been associated with maintained mtDNA content and seems to contribute to longevity [51]. However, the molecular mechanisms underlying the relationship among mitochondrial dynamics, senescence and longevity are not fully understood and deserve further investigation.

### 2.4. Autophagy and Mitophagy

**Figure 3:**
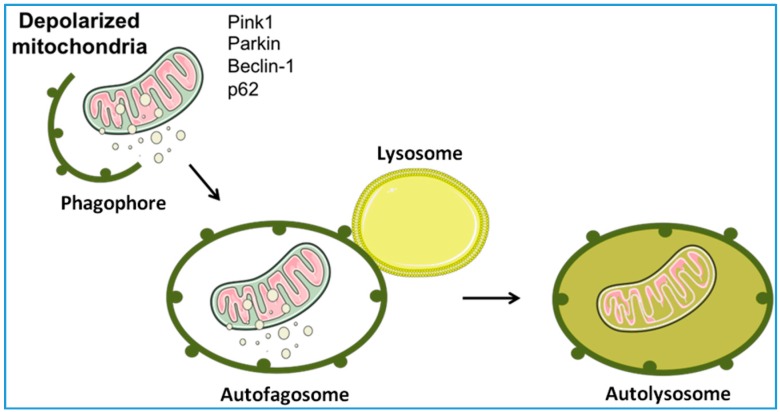
Schematic representation of mitophagy. Mitophagy ensures the selective degradation of dysfunctional mitochondria through specialized autophagy. The process begins with the formation of a double-layered membrane (phagophore) around the organelles to be degraded. By growing in size, the phagophore progressively engulfs the cargo, forming an autophagosome. The subsequent autophagosome fusion with lysosomes generates an autolysosome wherein the cargo is digested. PINK1, PTEN-induced putative kinase 1.

Autophagy is, literally, a cellular self-eating process through which intracellular components are degraded within lysosomes during periods of stress, such as nutrient deprivation, as an attempt to adapt and survive [52]. Selective autophagic removal of mitochondria (mitophagy) is triggered by the loss of mitochondrial membrane potential [53] and is aimed at limiting reactive oxygen species (ROS) generation and preserving cell viability through the clearance of dysfunctional organelles [54]. Mitophagy, however, represents an extreme attempt of the cell to maintain homeostasis since mitochondria can also take an alternative route to dispose damaged components, before whole-sale organelle degradation is triggered. Indeed, matrix components can be eliminated within vesicles budding from dysfunctional but not yet depolarized mitochondria [55,56]. Mitochondrion-derived vesicles (MDVs) serve to eliminate oxidized mitochondrial elements through the serine/threonine-protein kinase PTEN-induced putative kinase 1 (PINK1) and the E3 ubiquitin ligase Parkin [57]. As part of MQC, the delivery of damaged cargo within MDVs to lysosomes occurs as an early response to oxidative stress [55,56,57]. Conversely, severely damaged mitochondria are fissioned and targeted for elimination through a distinct pathway involving the synergistic activity of the mitochondrial dynamics machinery, PINK1, Parkin, Bnip3L/Nix, and Bnip3 [58] (Figure 3). Indeed, following mitochondrial depolarization, PINK1 accumulates on the mitochondrial surface, leading to the recruitment of Parkin, which ubiquitinates proteins located in the outer mitochondrial membrane [59]. Ubiquitination of the PARL protease promotes the execution of mitophagy by preventing PINK1 degradation. Ubiquitin-tagged mitochondria bind to p62, which assists in the recruitment of autophagosomal membranes to mitochondria [60]. Parkin can also interact with activating molecule in Beclin1-regulated autophagy (AMBRA1), which stimulates the activity of the class III phosphatidylinositol 3-kinase (PI3K) complex required for phagophore formation [61].

Dysfunctional vesicle trafficking as well as specific molecular patterns recruited in vesicle cargos has been described under several conditions [14,62]. A senescence-associated secretory phenotype has also been identified and characterized [63]. Studies have investigated the role of extracellular vesicles as carriers of senescence signals outside the cell [64]. Noticeably, mitochondrial dysfunction has been indicated among the mechanisms promoting the development of an aging phenotype [65]. However, the molecular identity of the factors involved is presently unknown and is the subject of active investigation.

## 3. Inflammation and Oxidative Stress

Inflammation has long been considered to be a defense response against microbial agents. It is now clear that a (chronic) inflammatory response can also occur in the absence of infections, a condition referred to as “sterile inflammation” [66]. The term “inflamm-aging” has been coined to indicate the chronic systemic inflammation status that develops during aging [67]. Although inflamm-aging has been associated with increased morbidity and mortality [68], the finding of high levels of pro-inflammatory markers in centenarians makes its detrimental effect questionable [69].

The anti-microbial inflammatory response and sterile inflammation are alternatively mounted through the recruitment of distinct macrophage subsets: (1) tissue-resident macrophages, which serve anti-inflammatory functions to safeguard tissue homeostasis and resolve local inflammation; and (2) circulating monocytes, which are pro-inflammatory and limit the spread of the infection [70].

Mitochondria play a central role in sterile inflammation through the activation of several pathways [71]. A redox-sensitive inflammatory signaling pathway involves mitochondrial calcium handling, ROS production, and nuclear factor κB (NF-κB) activation. Under calcium overload, such as burn injury or sepsis [72], the electron transport chain (ETC) becomes dysfunctional and elevated ROS generation occurs. Such a ROS burst represents a major pro-inflammatory stimulus through the modulation of the expression and activity of NF-κB [73].

Particularly interesting are the differential outcomes of redox imbalance in inflammation. In the setting of moderate inflammation, if cellular repair systems are overwhelmed, the intrinsic apoptotic cascade may be triggered [74]. In the context of severe inflammation, mitochondrial dysfunction and ROS-induced damage may instead drive necrosis, leading to the release of cellular contents, including whole and fragmented mitochondria [74].

**Figure 4:**
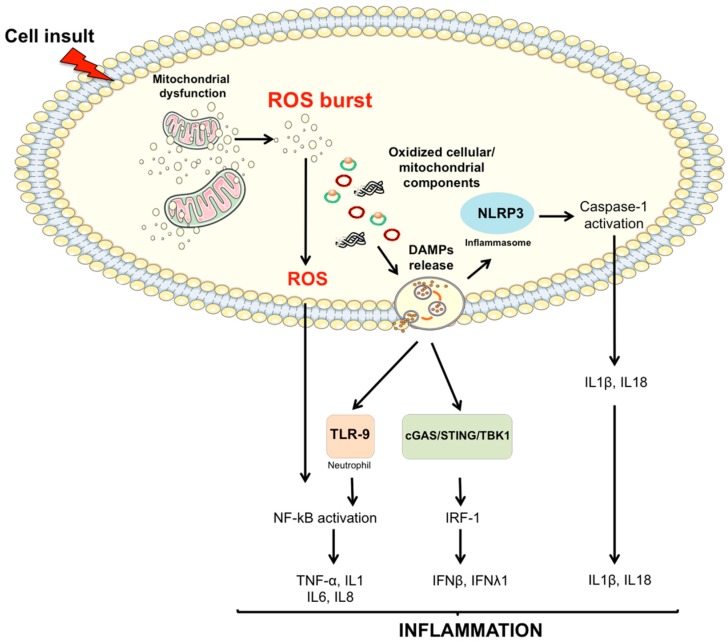
Proposed signaling pathways through which damaged-associated molecular patterns (DAMPs) can trigger inflammation. The impairment of mitochondrial quality control processes may lead to the accumulation of intracellular oxidized components and their release as DAMPs. Damaged mtDNA molecules, either TFAM-bound (green circles) or unbound (red circles) may be released as DAMPs. These, in turn, can activate an inflammatory response via three distinct signaling pathways by interacting with (1) Toll-like receptors (TLRs), (2) nucleotide-binding oligomerization domain (NOD)-like receptor family pyrin domain containing 3 (NLRP3) inflammasome, and (3) cytosolic cyclic GMP-AMP synthase (cGAS)-stimulator of interferon genes (STING) DNA-sensing system. IFN, interferon; IL, interleukin; IRF-1, interferon regulatory factor 1; mtDNA, mitochondrial DNA; NF-κB, nuclear factor κB; ROS, reactive oxygen species; TBK1, TANK-binding kinase 1; TNF-α, tumor necrosis factor α.

Another pathway through which mitochondria contribute to sterile inflammation is deeply rooted into the “danger theory” of inflammation proposed by Matzinger [75]. This pathway involves the accumulation of DAMPs released from injured cells [76]. DAMPs, in turn, induce caspase-1 activation and the release of pro-inflammatory cytokines [77]. Among the molecules listed as DAMPs, cell-free mtDNA, N-formyl peptides and cardiolipin are released from mitochondria in response to cell damage and death, and can activate inflammation [78,79]. Noticeably, degraded mtDNA has recently been identified as a DAMP subtype and a possible trigger of neurodegeneration [80]. Due to their bacterial ancestry, mitochondrial DAMPs can bind and activate membrane- or cytoplasmic-PRRs similar to those recognized by pathogen-associated molecular patterns (PAMPs) [15]. In particular, mtDNA activates different inflammatory responses through the binding of three different sensors: TLR, NLR, and cGAS-STING (Figure 4).

The TLR pathway is triggered by the binding of DAMPs to neutrophils, followed by their activation and subsequent organization of the inflammatory response via NF-κB signaling [81] (Figure 4). An alternative mtDNA-induced pathway operates through the NLR family pyrin domain containing 3 (NLRP3) inflammasome [82,83]. Given its association with several inflammatory diseases, NLRP3 has been one of the most studied NLRs. NLRP3 consists of a group of cytosolic protein complexes, the activation of which results in the engagement of caspase-1 [77]. The latter subsequently cleaves and activates interleukin (IL) 1β and 18 (Figure 4). It is worth noting that redox-sensitive inflammatory and inflammasome-mediated pathways may act synergistically to reinforce the inflammatory response [84].

The cGAS-STING DNA-sensing pathway is an additional component of the innate immune system [85]. Upon binding to mtDNA, cGAS proceeds through STING protein recruitment which triggers the phosphorylation of the transcription factor interferon regulatory factor 3 (IRF-3) via TANK-binding kinase (TBK). Activated IRF-3 induces the production of type I and III interferons (IFNs) (β and λ1) and IFN-stimulated nuclear gene products (Figure 4). A persistent inflammatory trigger is able to alert circulating immune cells, which, in turn, may mount a systemic response through the activation of mtDNA-induced inflammatory pathways. Cytokines, chemokines, nitric oxide (NO) and ROS released in the circulation by inflammatory cells can induce further mitochondrial damage, thereby establishing a vicious circle which reinforces the whole process.

Beside a generalized pro-oxidant environment, aging is also characterized by a decline in immune cell subtypes and functions, a condition referred to as immunosenescence [86]. Aged immune cells are an additional relevant source of ROS as they use an oxidative burst to carry out their defense functions. Interestingly, these cells are also targets of such molecules. In particular, age-related oxidative stress damages immune cells even more than others because of their peculiar membrane composition [87]. Interestingly, this condition, indicated as oxi-inflamm-aging, has been proposed to act via DAMP signaling [88].

## 4. Mitochondrial DAMPs: Waste-Derived Pro-Inflammatory Molecules

Ever since Matzinger proposed the “danger theory” of inflammation [75], several conditions characterized by an inflammatory response (e.g., trauma, HIV, cancer) have been found to be associated with increased levels of circulating mitochondrial DAMPs [13,78,89].

Interestingly, circulating levels of mtDNA molecules increase progressively past the age of 50 and correlate with those of pro-inflammatory cytokines, including IL6, tumor necrosis factor α (TNF-α), Regulated on Activation Normal T Cell Expressed and Secreted (RANTES), and IL1 receptor antagonist [90]. Remarkably, an increase in TNF-α production has been shown to occur when exposing monocytes to mtDNA concentrations similar to those detected in vivo, suggesting that circulating mtDNA may contribute to inflamm-aging [90].

TFAM has also been suggested to act as a mitochondrial DAMP [38]. Mouse embryonic fibroblasts expressing only one TFAM allele show a 50% decrease in mtDNA content associated with constitutive activation of the cGAS-STING-IRF-3 pathway [91]. The modulation of TFAM binding to mtDNA seems to be relevant during inflammation, as suggested by the involvement of TFAM in rerouting oxidized mtDNA to lysosomes for degradation in neutrophils [14]. Indeed, the extrusion of oxidized nucleoids by neutrophils in systemic lupus erythematosus is a powerful immune system activator [14]. Moreover, TFAM acts as a specific DAMP-inducing pro-inflammatory and cytotoxic response in in vitro models of human brain microglia [38].

General autophagy and mitophagy modulate inflammation by either clearing apoptotic corpses through macrophage activity or inhibiting NLRP3 inflammasome activation. Down-regulation of mitophagy results in spontaneous inflammasome activation as a consequence of mitochondrial ROS burst [82,92]. Accordingly, autophagy-deficient cells show accumulation of abnormal mitochondria characterized by increased levels of ROS and reduced membrane potential [92]. Recently, the activation of caspase-1 by NLRP3 has been shown to block mitophagy, thus reducing the clearance of damaged mitochondria and, as a positive feedback response, enhancing inflammasome activation [93]. These findings strongly support the existence of a pathway in which NLRP3 responds to mitochondrial dysfunction. More specifically, the accumulation of severely damaged mitochondria due to defective mitophagy could result in the extrusion of components able to induce NLRP3-mediated inflammation (Figure 4).

Cell death has been suggested to be another modulator of immune response through mitochondrial involvement and inflammasome activation [85]. ATP release following cell death has been proposed to function as a danger signal implicated in NLRP3 inflammasome activation [80]. Moreover, changes in mtDNA and TFAM content as well as alterations of the expression of autophagy-related genes have been found in several mitophagy dysfunction-associated conditions. Oxidative modifications occurring at the level of TFAM or mtDNA are indicated as major elements affecting TFAM binding and resulting in nucleoid instability. Both cell-free mtDNA and TFAM-bound mtDNA can act as DAMPs and elicit a systemic inflammatory response [14]. Accordingly, oxidized HMGB1, a member of the high mobility group box family of DNA, released by necrotic cells, is able to trigger a powerful immune response [94]. The potential role of oxidized TFAM both in the modulation of its binding to mtDNA and the inflammatory *milieu* of aging represents an uninvestigated but interesting scenario.

Despite the evidence supporting cell-free mtDNA and TFAM-bound mtDNA as DAMPs, the exact mechanisms of mtDNA delivery into the cytosol and then into the circulation are currently unknown. As elegantly reviewed by Safdar et al. [95], one of the mechanisms through which eukaryotic cells communicate with each other is a protein-based signaling system relying on exocytosis of proteins containing secretion-targeting sequences. Being too labile within the extracellular environment, proteins and other macromolecules (including mtDNA) may be secreted within small membranous extracellular vesicles [96]. A system of vesicles called exosomes is thought to use such a pathway to release a set of molecules (exerkines) in muscle under endurance exercise [96]. Exerkines contribute to mediating the beneficial effect of exercise by allowing systemic adaptations through autocrine, paracrine and/or endocrine signaling [95]. Interestingly, cell-free mtDNA has been identified among the molecules released within exosomes [97]. Although the actual mechanisms generating and releasing DAMPs is to date still unclear, their accumulation has been shown to activate tissue resident macrophages and favor tissue leukocyte infiltration [98].

## 5. Conclusions and Future Perspectives

Population aging poses a tremendous burden on the society. This has instigated intense research on the mechanisms that make the elderly more susceptible to diseases and disability. Several processes have been identified. Among these, inflamm-aging, a condition of chronic inflammation that develops independent of infections, has gained special attention. The cellular mechanisms responsible for inflamm-aging are not fully understood. However, recent studies suggest that a danger cellular-driven response may represent a relevant player. The coexistence of oxidative stress resulting from mitochondrial dysfunction and sterile inflammation has been summarized in the concept of oxy-inflamm-aging that merges the role of inflammation and oxidative stress in the aging process. Specific “danger molecules” generated in an oxidative *milieu* have been proposed to contribute to inflamm-aging. From this perspective, aging may be envisioned as the result of an “autoimmune-like” process. Given the role played by mitochondrial DAMPs in the activation of sterile inflammation, the mechanisms favoring organelle damage, in particular failing MQC processes, represent a relevant matter to be addressed by future investigations. The elucidation of these mechanisms may provide clinicians with novel therapeutics to counteract inflamm-aging and its negative correlates.

## Abbreviations

AMBRA1	activating molecule in beclin1-regulated autophagy
AMPK	AMP-activated protein kinase
cGAS	cytosolic cyclic GMP-AMP synthase
DAMPs	damage-associated molecular patterns
Drp1	dynamin-related protein 1
ER	endoplasmic reticulum
ETC	electron transport chain
Fis	fission protein 1
HMG	high-mobility-group
IFN	interferon
IL	interleukin
IRF-3	interferon regulatory factor 3
MDVs	mitochondrion-derived vesicles
Mfn	mitofusin
MQC	mitochondrial quality control
mtDNA	mitochondrial DNA
NF-kB	nuclear factor kB
NLR	nucleotide-binding oligomerization domain (NOD)-like receptor
NLRP3	NLR family pyrin domain containing 3
NO	nitric oxide
NR	nuclear respiratory factor
OPA1	optic atrophy protein 1
PAMPs	pathogen-associated molecular patterns
PARL	presenilins-associated rhomboid-like protein
PGC	peroxisome proliferator activated receptor gamma coactivator
PI3K	phosphatidylinositol 3-kinase
PINK	PTEN-induced putative kinase 1
PRRs	pattern recognition receptors
RANTES	Regulated on Activation Normal T Cell Expressed and Secreted
ROS	reactive oxygen species
SLE	systemic lupus erythematosus
STING	stimulator of interferon genes
TBK	TANK-binding kinase
TFAM	mitochondrial transcription factor A
TFBM	mitochondrial transcription factors B
TLR	Toll-like receptor
TNF-α	tumor necrosis factor α
TWEAK	TNF-like weak inducer of apoptosis
UPRmt	mitochondrial unfolded protein response
UPS	ubiquitin-proteasome system
